# Levels and predictors of anxiety, depression and health anxiety during COVID-19 pandemic in Turkish society: The importance of gender

**DOI:** 10.1177/0020764020927051

**Published:** 2020-05-08

**Authors:** Selçuk Özdin, Şükriye Bayrak Özdin

**Affiliations:** 1 Psychiatry Clinic, Faculty of Medicine, Ondokuz Mayıs University, Samsun, Turkey; 2 Rasathane Family Health Center, Samsun, Turkey

**Keywords:** Health anxiety, depression, anxiety, COVID-19

## Abstract

**Background:**

The COVID-19 pandemic is having negative effects on societies’ mental health. Both the pandemic and the measures taken to combat it can affect individuals’ mental health.

**Aims:**

The purpose of this study was to evaluate the levels of depression, anxiety and health anxiety in Turkish society during the COVID-19 pandemic, and to examine the factors affecting these.

**Method:**

The study was performed using an online questionnaire. Participants were asked to complete a sociodemographic data form, the Hospital Anxiety and Depression Scale (HADS) and the Health Anxiety Inventory (HAI). The effects on depression, anxiety and health anxiety levels of factors such as age, sex, marital status, living with an individual aged above 60, the presence of a new Coronavirus+ patient among friends or relatives, previous and current psychiatric illness and presence of accompanying chronic disease were then investigated.

**Results:**

In terms of HADS cut-off points, 23.6% (*n* = 81) of the population scored above the depression cut-off point, and 45.1% (*n* = 155) scored above the cut-off point for anxiety. In regression analysis, female gender, living in urban areas and previous psychiatric illness history were found as risk factors for anxiety; living in urban areas was found as risk factor for depression; and female gender, accompanying chronic disease and previous psychiatric history were found as risk factors for health anxiety.

**Conclusion:**

The results of this cross-sectional study suggest that the groups most psychologically affected by the COVID-19 pandemic are women, individuals with previous psychiatric illness, individuals living in urban areas and those with an accompanying chronic disease. Priority might therefore be attached to these in future psychiatric planning.

## Background

A novel Coronavirus type (2019-nCoV) was identified as an etiological agent in cases of pneumonia of indefinite cause seen in the Chinese city of Wuhan on 31 December 2019 (Zhu et al., 2020). The virus subsequently spread rapidly across the world and led to the pandemic known as COVID-19. Although the virus was first seen in China, countries in the European and American continents are the most affected. The first case in Turkey was reported on 11 March 2020. Various precautions began being adopted as the number of cases increased, and a wide range of measures are still in place. As of 20 April 2020, the total number of reported cases in Turkey was 90,980, with 2,140 deaths (Republic of Turkey Ministry of Health, 2020).

Traumatic events can reduce people’s feeling of security, remind them of the fact of death and have adverse effects on their mental health. Questions related to the pandemic with no definite answers, such as when it will come to an end and methods of treatment; constant exposure to a flow of information about the pandemic and its effects; decreased social relations due to the pandemic; and recommendations/prohibitions such as remaining at home as much as possible all can adversely affect individuals’ mental health. Symptoms such as anxiety, depression, fear, stress and sleep problems are being seen more frequently during the COVID-19 pandemic (Torales et al., 2020). An incidence of depression, anxiety and post-traumatic stress disorder symptoms between 10% and 18% was reported during and after the Severe Acute Respiratory Syndrome (SARS) epidemic (Wu et al., 2005). One study of 253 individuals from one of the regions most affected by the COVID-19 pandemic in China reported a 7% incidence of post-traumatic stress symptoms 1 month after the outbreak of the pandemic (Liu et al., 2020). Another study from China observed that 53% of people experienced feelings of terror (Zhang & Ma, 2020). One extensive study determined that 0.9% of university students exhibited severe symptoms of anxiety, 2.7% moderate symptoms and 21.3% mild symptoms. Family income stability and living together with parents were found to exhibit a protective effect against anxiety symptoms (Cao et al., 2020). Variables such as occupation, education and gender have been found to affect symptoms of anxiety and depression developing during the pandemic (Y. Wang et al., 2020).

Health anxiety is a multifaceted phenomenon, consisting of distressing emotions, physiological arousal and associated bodily sensations, thoughts and images of danger and avoidance and other defensive behaviors. The phenomenon is experienced on an occasional basis by many people in daily life. Health anxiety is a crucial issue, and both increases and decreases can result in problems (GjG, 2004). Considering heath anxiety as a broad spectrum, individuals’ health anxiety can be classified as high or low (Taylor, 2019). Some individuals with high health anxiety during the pandemic may cause crowding in the health system by presenting to physicians and hospitals on a frequent basis. Others with high anxiety may be reluctant to seek medical assistance out of concerns that hospitals are sources of transmission. In contrast, individuals with low health anxiety may be reluctant to comply with warnings regarding bringing the pandemic under control, and may behave in a highly relaxed manner (Asmundson & Taylor, 2020). One study investigating the number of visits to 18 emergency departments in the United States following the H1N1 pandemic showed that the emergency department visits in the week before the pandemic reached the country was as high as the number in the week when the pandemic was most intense. The number of presentations was also 7% higher than in normal times (McDonnell et al., 2012). The ability to bring pandemics under control depends on compliance with warnings. One psychological factor capable of affecting adherence to warnings is health anxiety.

The current COVID-19 pandemic and the measures adopted against it are as follows: The psychological effects on the Turkish public may vary depending on variables such as gender, place of residence, age, accompanying chronic disease, previous or current psychiatric illness and presence of a COVID+ relative. Since anxiety and depressive disorders are more prevalent in women (Alexander et al., 2007), women are also estimated to be more affected during pandemics (Kim et al., 2014). Crowded areas involve a greater risk for droplet-transmitted 2019-nCoV. Individuals living in urban areas may therefore also be expected to be more affected. As is known from previous epidemics, individuals with a history of current or previous psychiatric illness are significantly affected (Page et al., 2011). 2019-nCoV is known to exhibit a particularly severe course in the above 60s and individuals with accompanying chronic disease (Zhou et al., 2020). This group and individuals living with someone above 60 may therefore be expected to be more severely affected.

To the best of our knowledge, no previous studies have investigated health anxiety during the COVID-19 pandemic. This research investigated the levels of depression, anxiety and health anxiety in Turkish society during the COVID-19 pandemic. This study also investigated the effect on these variables of potentially affecting factors such as age, gender, place of residence, accompanying chronic disease, a COVID+ friend or relative, living with someone aged above 60 and a current or previous history of psychiatric disease.

## Method

The research population in this descriptive population consisted of individuals aged above 18 living in various provinces of Turkey. Minitab 19.0 software was used to determine the sample size. The sample size was calculated based on an expected prevalence of 6.3% (Y. Wang et al., 2020), Type 1 error of 5% and study power of 95% based on a similar previous study. The calculation showed that at least 159 individuals would need to be enrolled. However, to be able to perform subgroup comparisons within the main study group, we planned to enroll 318 participants. Data were collected through an online questionnaire between 14 and 16 April 2020, using SurveyMonkey software (SurveyMonkey, San Mateo, CA, USA). Individuals agreeing to participate were asked to complete the questionnaire through social media (WhatsApp, Twitter and Facebook). The 54-item questionnaire consisted of a sociodemographic data form, the Hospital Anxiety and Depression Scale (HADS) and the Health Anxiety Inventory (HAI).

HADS is a self-report scale developed by Zigmond and Snaith (1983) which is used to determine anxiety and depression levels. It consists of 14 questions, each of which is scored 0–3. Anxiety and depression are evaluated with seven questions each. The lowest possible scores for depression and anxiety are 0, and the highest possible scores are 21. Higher scores indicate increased severity of anxiety or depression. The reliability and validity of the Turkish language version were examined by Aydemir et al. Cut-off scores for Turkish society have been determined as 7 for anxiety and 10 for depression (Aydemir et al., 1997).

HAI is a self-report scale developed by Salkovskis et al. (2002) and contains 18 questions. The first 14 questions consist of four options from which individuals select the one that best describes their mental state. The last four questions are intended to measure mental state in the event of severe disease. All questions are scored 0–3, with higher scores indicating greater health anxiety. The total score ranges from 0 to 54. The validity and reliability were studied by Aydemir et al. (2013).

Statistical analyses were performed on SPSS 15.0 software. Results were expressed as mean ± standard deviation or number (%). The Kolmogorov–Smirnov test was used to assess normal distribution of quantitative data. The use of nonparametric tests at data analysis was found to be appropriate. The Mann–Whitney *U*-test was applied in two-way group comparisons, and the chi-square test for quantitative data. Participants were grouped as individuals with anxiety and depression based on HADS cut-off scores, and binary logistic regression was used to identify factors associated with anxiety and depression. Multiple linear regression analysis was used to identify the factors associated with health anxiety. Due to the absence of sufficient individuals aged above 60 (*n* = 9), a cut-off point of 50 was determined. Values of *p* less than .05 were regarded as significant for all tests. Approval for the study was granted by the Ondokuz Mayıs University Clinical Research Ethical Committee (no. 2020/147).

## Results

A total of 343 individuals completed the questionnaire online, 278 of whom lived in an urban area and 71 (20.9%) of whom lived with another person aged above 60. Participants shared their homes with a mean 3.51 people (±1.27). Overall, 250 (72.8%) participants were working before the pandemic, of whom 89 stopped or suspended working after it; 35 participants (10.2%) had friends or relatives with COVID-19; 54 (15.7%) participants had a chronic disease; and 62 (42.5%) of the 162 individuals who smoked before the pandemic reported quitting or cutting down after it. The number of participants who had gone outside in the previous week was 282 (82.2%), the most common reasons cited being to buy food (*n* = 224) and for work (*n* = 118). And 15 (4.3%) participants did not follow news stories or developments about COVID-19. Participants’ mean HADS anxiety and depression scores were 6.8 ± 4.2 and 6.7 ± 4.2, respectively. HAI total score was 15.1 ± 7.0 (Table 1).

**Table 1.** Sociodemographic and clinical features of the participants.

Variables					
Age (years), *M* ± *SD*	37.16 ± 10.31				
Gender, *n* (%)	Male	174 (50.7)			
	Female	169 (49.2)			
Marital status, *n* (%)	Married	218 (63.5)			
	Single^a^	125 (36.4)			
Place of residence, *n* (%)	Urban	278 (81.0)			
	Rural	65 (18.9)			
Education time, *M* ± *SD*	14.85 ± 3.95				
Working before pandemic, *n* (%)	Yes	250 (72.8)	Working after pandemic	Yes	161 (64.4)
				No	89 (35.6)
	No	93 (27.1)			
No. of people living together, *M* ± *SD*	3.51 ± 1.27				
Living with an individual aged above 60, *n* (%)	Yes	72 (20.9)			
	No	271 (79.0)			
Friends or relatives with COVID, *n* (%)	Yes	35 (10.2)			
	No	308 (89.8)			
Accompanying chronic disease, *n* (%)	Yes	54 (15.7)			
	No	289 (84.3)			
Smoking, *n* (%)	No	181 (52.7)			
	Yes, with the same frequency	79 ( 23.0)			
	Yes, reduced	69 (20.1)			
	Yes, increased	14 (4.0)			
Previous or current psychiatric illness, *n* (%)	Yes	75 (21.8)			
	No	268 (78.2)			
History of going out in the past week, *n* (%)	Yes	282 (82.2)			
	No	61 (17.7)			
Reason for going out in the previous week, *n*	Work	118			
	Food supply	224			
	Health	68			
	Financial transactions	60			
	Others	46			
Following new stories about COVID-19, *n* (%)	Yes	324 (95.7)			
	No	15 (4.3)			
HADS, *M* ± *SD*	Anxiety subscale	6.8 ± 4.2			
	Depression subscale	6.7 ± 4.2			
HAI, *M* ± *SD*	Total	15.1 ± 7.0			

HADS: Hospital Anxiety and Depression Scale; HAI: Health Anxiety Inventory.

a Divorced and widowed people are grouped together with singles because there are few.

Depression scores were significantly higher among women, individuals living in an urban area, individuals with COVID+ patients among friends or relatives, individuals with current or previous psychiatric illness history and individuals with chronic disease. Anxiety scores were significantly higher among women, individuals with a COVID+ patient among friends and relatives and individuals with a current psychiatric disease. Total HAI score was significantly higher among women, individuals with current or previous psychiatric illness and individuals with chronic disease (Table 2). In terms of HADS cut-off points, 23.6% (*n* = 81) of the population scored above the depression cut-off point, and 45.1% (*n* = 155) scored above the cut-off point for anxiety.

**Table 2.** Comparison of the participants divided into groups in terms of HADS and HAI.

Variables		HADS		HAI *M* ± *SD*
		Depression *M* ± *SD*	Anxiety *M* ± *SD*	
Gender	Male	6.2 ± 3.8	5.9 ± 3.6	14.2 ± 6.2
	Female	7.2 ± 4.4	7.7 ± 4.5	15.9 ± 7.6
	*p*-value	.047*	.000*	.030
Age groups	18–49	6.8 ± 4.1	6.8 ± 4.1	15.1 ± 6.8
	⩾50	6.14 ± 4.9	6.3 ± 4.7	14.7 ± 8.0
	*p*-value	.171	.310	.458
Marital status	Married	6.4 ± 4.0	6.6 ± 4.0	14.5 ± 6.4
	Single^a^	7.2 ± 4.4	7.0 ± 4.4	16.1 ± 7.8
	*p*-value	.091	.664	.144
Place of residence	Urban	6.9 ± 4.2	6.9 ± 4.3	15.2 ± 7.1
	Rural	5.8 ± 4.1	6.2 ± 3.3	14.4 ± 6.3
	*p*-value	.029*	.373	.550
Working after pandemic	Yes	7.0 ± 4.2	6.9 ± 4.3	14.8 ± 6.7
	No	6.3 ± 3.8	6.2 ± 3.7	14.1 ± 6.3
	*p*-value	.340	.308	.542
Living with an individual aged above 60	Yes	7.7 ± 4.3	6.7 ± 4.1	14.6 ± 7.7
	No	6.6 ± 4.1	6.8 ± 4.2	15.2 ± 6.8
	*p*-value	.433	.952	.403
Friends or relatives with COVID	Yes	9.0 ± 4.6	8.4 ± 4.4	16.8 ± 7.7
	No	6.4 ± 4.0	6.6 ± 4.1	14.9 ± 6.9
	*p*-value	.001*	.014*	.144
Previous psychiatric illness	Yes	8.3 ± 4.8	7.8 ± 4.7	18.3 ± 7.2
	No	6.5 ± 4.1	6.7 ± 4.1	14.8 ± 6.9
	*p*-value	.036*	.206	.011*
Current psychiatric illness	Yes	7.9 ± 4.6	8.5 ± 4.8	18.0 ± 8.2
	No	6.4 ± 4.0	6.3 ± 3.9	14.3 ± 6.4
	*p*-value	.020*	.000*	.000*
History of going out in the previous week	Yes	6.8 ± 4.2	6.9 ± 4.2	15.0 ± 7.0
	No	6.1 ± 4.0	6.2 ± 4.0	15.5 ± 6.8
	*p*-value	.247	.369	.342
Accompanying chronic disease	Yes	7.8 ± 4.5	7.7 ± 4.3	17.9 ± 7.6
	No	6.5 ± 4.1	6.6 ± 4.1	14.6 ± 6.7
	*p*-value	.050*	.061	.001*

HADS: Hospital Anxiety and Depression Scale, HAI: Health Anxiety Inventory.

a Divorced and widowed people are grouped together with singles because there are few.

* *p* < .05.

According to the results of multiple binary logistic regression analysis evaluating the risk factors for depression and anxiety, living in urban areas (OR = 0.534, 95% CI = (0.297, 0.960)) was found to be a risk factor for depression, while female gender (OR = 2.478, 95% CI = (1.439, 4.267)), living in urban areas (OR = 0.362, 95% CI = (0.159, 0.823)) and previous psychiatric illness (OR = 0.363, 95% CI = (0.196, 0.675)) were identified as risk factors for anxiety (Table 3). According to the results of multiple linear regression analysis evaluating risk factors for health anxiety, female gender (β = .105, *p* = .047), accompanying chronic disease (β = .160, *p* = .003) and previous psychiatric illness (β = .176, *p* = .001) were found to predict health anxiety (Table 4).

**Table 3.** Results of logistic regression analysis on factors significantly associated with depression and anxiety.

	According to HADS anxiety vs non anxiety		According to HADS depression vs non-depression	
	OR (95% CI)	*p*-value	OR (95% CI)	*p*-value
Gender (female vs male)	2.478 (1.439, 4.267)	.001*	1.298 (0.833, 2.022)	.249
Place of residence (urban vs rural)	0.362 (0.159, 0.823)	.015*	0.534 (0.297, 0.960)	.036*
Friends or relatives with COVID (yes vs no)	0.650 (0.297, 1.423)	.281	0.485 (0.232, 1.012)	.054
Accompanying chronic disease (yes vs no)	0.601 (0.302, 1.197)	.148	0.587 (0.317, 1.087)	.090
Current psychiatric illness (yes vs no)	0.981 (0.388, 2.484)	.968	1.002 (0.429, 2.340)	.996
Previous psychiatric illness (yes vs no)	0.363 (0.196, 0.675)	.001*	0.573 (0.323, 1.019)	.058

HADS: Hospital Anxiety and Depression Scale; OR: odds ratio; CI: confidence interval.

* *p* < .05.

**Table 4.** Results of multiple linear regression analysis^a^ of clinical variables for predicting HAI subscales.

	HAI			
	*B*	β	95% CI for *B*	*p*-value
Gender (female vs male)	1.475	.105	(0.022, 2.928)	.047*
Place of residence (urban vs rural)	0.560	.031	(−1.291, 2.411)	.552
Friends or relatives with COVID (yes vs no)	1.476	.064	(−0.906, 3.858)	.224
Accompanying chronic disease (yes vs no)	3.133	.160	(1.098, 5.168)	.003*
Current psychiatric illness (yes vs no)	1.375	.054	(−1.403, 4.154)	.331
Previous psychiatric illness (yes vs no)	3.088	.176	(1.195, 4.982)	.001*

HAI: Health Anxiety Inventory; CI: confidence interval.

a Forced entry model was applied.

* *p* < .05.

## Discussion

This study investigated the levels of depression, anxiety and health anxiety in Turkish society during the COVID-19 pandemic and found that women, and individuals living in urban areas, with a COVID+ patient among friends or relatives, previously or currently in receipt of psychiatric treatment and with at least one accompanying chronic disease, were more severely affected.

Various measures are being taken during the pandemic to reduce the spread of the virus such as social distancing, lockdowns and self-isolation. At the same time, the number of patients contracting the disease and death rates continue to grow rapidly. Based on the number of reported cases, Turkey had the seventh highest number of reported cases as of 20 April 2020 (World Health Organization, 2020). All these factors can have adverse effects on the mental health of the society. The relatively high depression and anxiety levels and rates (23.6% and 45.1%, respectively) are therefore expected findings in terms of pandemic psychological effects. One extensive study from China reported that approximately 35% of people were psychologically affected by the pandemic (Qiu et al., 2020). The high incidence of depression and anxiety in individuals with previous histories of psychiatric illness may be a finding associated with recurrence of psychiatric diseases before and after the pandemic, as shown in previous such studies (Lee et al., 2007). People are being asked not to leave their homes during the pandemic. In addition, individuals with psychiatric symptoms experience difficulties in obtaining medical assistance for reasons such as some hospitals being converted into pandemic hospitals, psychiatric clinics being unable to provide active health services or number of patients examined being reduced for safety reasons, and hospital environments constituting a risk in terms of viral load.

Individuals experience varying levels of psychological distress during pandemics. A low level of health anxiety in pandemics may result in situations that adversely affect people’s lives from numerous perspectives, due to failure to comply with protective measures. It is therefore important for everyone to exhibit the same awareness and responsibility at times when collective awareness needs to be high, such as pandemics. This study shows that women, individuals with past or present psychiatric illnesses and individuals with chronic disease have greater sensitivity to and awareness of sensations in their own bodies. Health anxiety may be higher in women and individuals with a history of psychiatric disease (Bobevski et al., 2016). The increase in health anxiety in individuals with chronic disease linked to an increased risk may also reflect the psychological distress that people feel during the COVID-19 pandemic. As far as is known, the level of health anxiety was studied for the first time in the COVID-19 pandemic. It was found that female gender, accompanying chronic disease, and previous psychiatric illness history predict sensitivity to physical sensations.

In this study, depression, anxiety and health anxiety levels were higher in women, showing that the psychiatric impact during the COVID-19 pandemic may be greater on women. Several previous studies have shown that anxiety disorders and depressive disorders are more frequent in women (Alexander et al., 2007). Female gender has been identified as the most potent predictor of post-traumatic stress disorder symptoms after pandemics (Liu et al., 2020). In a study from China, although women were better informed about the disease than men during the COVID-19 pandemic and complied more with advice, such as wearing masks and avoiding public spaces, they also reported not knowing whether the pandemic could be brought under control or the probability of such control being established (Zhong et al., 2020). Anxiety disorder has been seen at three-fold higher levels in women than in men during the COVID-19 pandemic (Y. Wang et al., 2020). High health anxiety may result in the individual misinterpreting his or her own sensations, and may leave the individual vulnerable to negative affective states such as anxiety and depression. In light of our present knowledge, the higher levels of anxiety, depression and health anxiety in women in this study is not an unexpected finding.

2019-nCoV is a viral agent mainly transmitted through droplets or direct contact. Such viruses can be transmitted more, and more easily, in urban and central areas with denser human populations (Taylor, 2019). Psychological impact may therefore be greater in individuals in urban areas (Chen et al., 2020). Individuals living in urban areas may also have a greater probability of access to communication and information. The majority of COVID-19 cases in Turkey are known to be in urban areas. The higher levels of depression among people living in urban areas, where the probabilities of encountering the virus are also higher, is therefore also an expected finding.

Advanced age and comorbid chronic diseases have been identified as the most important risk factors for mortality due to 2019-nCoV (Zhou et al., 2020). In addition, the elderly and individuals with chronic disease have an increased risk of contracting the disease (B. Wang et al., 2020). Parallel to all these findings, increased levels of health anxiety, depression and anxiety were also observed in individuals with chronic disease. However, we found no statistically significant difference in terms of depression, anxiety and health anxiety levels between individuals living with another person aged above 60 and the control group. Although this is a difficult finding to account for, participants’ relatively high educational level (14.85 ± 3.95) may have a protective effect against negative emotions (Y. Wang et al., 2020). Individuals with high educational levels have higher levels of information about and better attitudes toward COVID-19 (Zhong et al., 2020). At the same time, there was no significant difference in variables between the 18–49 and above-50 age groups. The 60-and-above age group is generally reported to be the group in which the course of disease is most severe; 80% of mortality is in this age group (CDC COVID-19 Response Team, 2020). To show the difference, the age cut-off value in this study (50 years) may have remained low. Another reason may be the protective role against life events of crystallized intelligence with increasing experience, as suggested by Cattell (1963).

Not following new stories about COVID-19 (*n* = 15, 4.3%) may be regarded as avoidance behavior. Many Turkish television channels are presenting news broadcasts and programs about the pandemic. At the same time, there is more content about the pandemic on the social media. Other factors in the social-psychological field of pandemic psychology are rumors and observational learning (Taylor, 2019). These also frequently occur during the COVID-19 pandemic. Avoidance is a defense mechanism that can be used as a means of coping with anxiety. The prevalence of avoidance behavior during the swine flu epidemic in the United Kingdom in 2009 was reported as 4.9% (Rubin et al., 2009). Avoidance has been identified as one of the behavioral coping methods employed during outbreaks (Chew et al., 2020). The rate of avoidance in this study is therefore unsurprising.

One particular advantage of this study is that it measured the public psychological state during the pandemic. Depression, anxiety and health anxiety levels were evaluated in a cross-sectional manner in the pandemic. One of the principal limitations of this study is that due to the cross-sectional nature of the research, it is difficult to draw any conclusions regarding its long-term effect. It is difficult to apply sampling methods at this time because of the pandemic. In addition, there is also the possibility of selection bias since the study was performed with an online questionnaire. Individuals without Internet and unable or unwilling to use smartphones or email could not be included in the study.

## Conclusion

In conclusion, our findings suggest that the pandemic may have a greater effect on women, individuals with a past or present psychiatric disease, individuals living in urban areas and individuals with a comorbid chronic disease. Priority might therefore perhaps be attached to psychological support measures for members of these groups.

## References

[bibr1-0020764020927051] Alexander J. L. Dennerstein L. Kotz K. Richardson G. (2007). Women, anxiety and mood: A review of nomenclature, comorbidity and epidemiology. Expert Review of Neurotherapeutics, 7(11 Suppl.), S45–S58. 10.1586/14737175.7.11s.S45 18039068

[bibr2-0020764020927051] Asmundson G. J. G. Taylor S. (2020). How health anxiety influences responses to viral outbreaks like COVID-19: What all decision-makers, health authorities, and health care professionals need to know. Journal of Anxiety Disorders, 71, 102211. 10.1016/j.janxdis.2020.102211 PMC7271220 32179380

[bibr3-0020764020927051] Aydemir O. Güvenir T. Küey L. Kültür S. (1997). Hastane Anksiyete ve Depresyon Ölçeği Türkçe Formunun Geçerlilik ve Güvenilirlik Çalışması [Reliability and Validity of the Turkish version of Hospital Anxiety and Depression Scale]. Turkish Journal of Psychiatry, 8, 280–287.

[bibr4-0020764020927051] Aydemir O. Kırpınar I. Sati T. Uykur B. Cengisiz C. (2013). Reliability and Validity of the Turkish Version of the Health Anxiety Inventory. Archives of Neuropsychiatry, 50, 325–331. 10.4274/npa.y6383 28360565 PMC5363424

[bibr5-0020764020927051] Bobevski I. Clarke D. M. Meadows G. (2016). Health anxiety and its relationship to disability and service use: Findings from a large epidemiological survey. Psychosomatic Medicine, 78(1), 13–25. 10.1097/psy.0000000000000252 26588821

[bibr6-0020764020927051] Cao W. Fang Z. Hou G. Han M. Xu X. Dong J. Zheng J. (2020). The psychological impact of the COVID-19 epidemic on college students in China. Psychiatry Research, 287, 112934. 10.1016/j.psychres.2020.112934 PMC7102633 32229390

[bibr7-0020764020927051] Cattell RB (1963). Theory of fluid and crystallized intelligence: A critical experiment. Journal of Educational Psychology, 54(1), 1–22. 10.1037/h0024654 6043849

[bibr8-0020764020927051] CDC COVID-19 Response Team. (2020). Severe outcomes among patients with Coronavirus Disease 2019 (COVID–19) – United States, 2 12-3 16, 2020 Morbidity and Mortality Weekly Report, 69(12), 343–346. 10.15585/mmwr.mm6912e2 32214079 PMC7725513

[bibr9-0020764020927051] Chen Y. Jin Y. L. Zhu L. J. Fang Z. M. Wu N. Du M. X.. . . Yao Y. S. (2020). [The network investigation on knowledge, attitude and practice about COVID-19 of the residents in Anhui Province]. Zhonghua Yu Fang Yi Xue Za Zhi, 54(4), 367–373. 10.3760/cma.j.cn112150-20200205-00069 32268643

[bibr10-0020764020927051] Chew Q. H. Wei K. C. Vasoo S. Chua H. C. Sim K. (2020). Narrative synthesis of psychological and coping responses towards emerging infectious disease outbreaks in the general population: Practical considerations for the COVID-19 pandemic. Singapore Medical Journal. Advance online publication. 10.11622/smedj.2020046 PMC7926608 32241071

[bibr11-0020764020927051] GjG T. S. A. (2004). Treating health anxiety. Guilford.

[bibr12-0020764020927051] Kim S. J. Han J. A. Lee T. Y. Hwang T. Y. Kwon K. S. Park K. S.. . . Lee S. Y. (2014). Community-based risk communication survey: Risk prevention behaviors in communities during the H1N1 crisis, 2010. Osong Public Health and Research Perspectives, 5(1), 9–19. 10.1016/j.phrp.2013.12.001 24955307 PMC4064644

[bibr13-0020764020927051] Lee A. M. Wong J. G. McAlonan G. M. Cheung V. Cheung C. Sham P. C.. . . Chua S. E. (2007). Stress and psychological distress among SARS survivors 1 year after the outbreak. Canadian Journal of Psychiatry, 52(4), 233–240. 10.1177/070674370705200405 17500304

[bibr14-0020764020927051] Liu N. Zhang F. Wei C. Jia Y. Shang Z. Sun L.. . . Liu W. (2020). Prevalence and predictors of PTSS during COVID-19 outbreak in China hardest-hit areas: Gender differences matter. Psychiatry Research, 287, 112921. 10.1016/j.psychres.2020.112921 PMC7102622 32240896

[bibr15-0020764020927051] McDonnell W. M. Nelson D. S. Schunk J. E. (2012). Should we fear ‘flu fear’ itself? Effects of H1N1 influenza fear on ED use. The American Journal of Emergency Medicine, 30(2), 275–282. 10.1016/j.ajem.2010.11.027 21208765

[bibr16-0020764020927051] Page L. A. Seetharaman S. Suhail I. Wessely S. Pereira J. Rubin G. J. (2011). Using electronic patient records to assess the impact of swine flu (influenza H1N1) on mental health patients. Journal of Mental Health, 20(1), 60–69. 10.3109/09638237.2010.542787 21271827

[bibr17-0020764020927051] Qiu J. Shen B. Zhao M. Wang Z. Xie B. Xu Y. (2020). A nationwide survey of psychological distress among Chinese people in the COVID-19 epidemic: Implications and policy recommendations. General Psychiatry, 33(2), Article e100213. 10.1136/gpsych-2020-100213 PMC7061893 32215365

[bibr18-0020764020927051] Republic of Turkey Ministry of Health. (2020). https://covid19.saglik.gov.tr/

[bibr19-0020764020927051] Rubin G. Amlôt R. Page L. Wessely S. (2009). Public perceptions, anxiety, and behaviour change in relation to the swine flu outbreak: Cross sectional telephone survey. BMJ, 339, Article b2651. 10.1136/bmj.b2651 PMC2714687 19574308

[bibr20-0020764020927051] Salkovskis P. M. Rimes K. A. Warwick H. M. Clark D. M. (2002). The Health Anxiety Inventory: Development and validation of scales for the measurement of health anxiety and hypochondriasis. Psychological Medicine, 32(5), 843–853. 10.1017/s0033291702005822 12171378

[bibr21-0020764020927051] Taylor S. (2019). The psychology of pandemics: Preparing for the next global outbreak of infectious disease. Cambridge Scholars Publishing.

[bibr22-0020764020927051] Torales J. O’Higgins M. Castaldelli-Maia J. M. Ventriglio A. (2020). The outbreak of COVID-19 coronavirus and its impact on global mental health. International Journal of Social Psychiatry. Advance online publication. 10.1177/0020764020915212 32233719

[bibr23-0020764020927051] Wang B. Li R. Lu Z. Huang Y. (2020). Does comorbidity increase the risk of patients with COVID-19: Evidence from meta-analysis. Aging, 12, 6049–6057. 10.18632/aging.103000 32267833 PMC7185114

[bibr24-0020764020927051] Wang Y. Di Y. Ye J. Wei W. (2020). Study on the public psychological states and its related factors during the outbreak of coronavirus disease 2019 (COVID-19) in some regions of China. Psychology, Health & Medicine. Advance online publication. 10.1080/13548506.2020.1746817 32223317

[bibr25-0020764020927051] Wu K. K. Chan S. K. Ma T. M. (2005). Posttraumatic stress, anxiety, and depression in survivors of severe acute respiratory syndrome (SARS). Journal of Traumatic Stress, 18(1), 39–42. 10.1002/jts.20004 16281194 PMC7166878

[bibr26-0020764020927051] Zhang Y. Ma Z. F. (2020). Impact of the COVID-19 pandemic on mental health and quality of life among local residents in Liaoning Province, China: A cross-sectional study. International Journal of Environmental Research and Public Health, 17(7), Article 2381. 10.3390/ijerph17072381 PMC7177660 32244498

[bibr27-0020764020927051] Zhong B.-L. Luo W. Li H.-M. Zhang Q.-Q. Liu X.-G. Li W.-T. Li Y. (2020). Knowledge, attitudes, and practices towards COVID-19 among Chinese residents during the rapid rise period of the COVID-19 outbreak: A quick online cross-sectional survey. International Journal of Biological Sciences, 16(10), 1745–1752. 10.7150/ijbs.45221 32226294 PMC7098034

[bibr28-0020764020927051] Zhou F. Yu T. Du R. Fan G. Liu Y. Liu Z.. . . Cao B. (2020). Clinical course and risk factors for mortality of adult inpatients with COVID-19 in Wuhan, China: A retrospective cohort study. The Lancet, 395(10229), 1054–1062. 10.1016/s0140-6736(20)30566-3 PMC7270627 32171076

[bibr29-0020764020927051] Zhu N. Zhang D. Wang W. Li X. Yang B. Song J.. . . Tan W. (2020). A Novel Coronavirus from Patients with Pneumonia in China, 2019. New England Journal of Medicine, 382(8), 727–733. 10.1056/NEJMoa2001017 31978945 PMC7092803

[bibr30-0020764020927051] Zigmond A. S. Snaith R. P. (1983). The hospital anxiety and depression scale. Acta Psychiatrica Scandinavica, 67(6), 361–370. 10.1111/j.1600-0447.1983.tb09716.x 6880820

